# The Molecular Effects of Environmental Enrichment on Alzheimer’s Disease

**DOI:** 10.1007/s12035-022-03016-w

**Published:** 2022-09-09

**Authors:** Anthony Kin Yip Liew, Chuin Hau Teo, Tomoko Soga

**Affiliations:** Jeffrey Cheah School of Medicine and Health Sciences, Monash University Malaysia, 47500 Bandar Sunway, Selangor Malaysia

**Keywords:** Environmental enrichment, Hippocampus, Molecular Pathways, Aging, Alzheimer’s Disease, Neurogenesis

## Abstract

Environmental enrichment (EE) is an environmental paradigm encompassing sensory, cognitive, and physical stimulation at a heightened level. Previous studies have reported the beneficial effects of EE in the brain, particularly in the hippocampus. EE improves cognitive function as well as ameliorates depressive and anxiety-like behaviors, making it a potentially effective neuroprotective strategy against neurodegenerative diseases such as Alzheimer’s disease (AD). Here, we summarize the current evidence for EE as a neuroprotective strategy as well as the potential molecular pathways that can explain the effects of EE from a biochemical perspective using animal models. The effectiveness of EE in enhancing brain activity against neurodegeneration is explored with a view to differences present in early and late life EE exposure, with its potential application in human being discussed. We discuss EE as one of the non pharmacological approaches in preventing or delaying the onset of AD for future research.

## Introduction

With the increase in life expectancy due to advances in technology, the elderly population is projected to double to 1.5 billion in the year 2050 [1]. As such, the issue of neurodegenerative diseases and dementia is a ticking time bomb which could implode as time progresses. Alzheimer’s disease (AD) is the most common cause of dementia amongst this slew of neurodegenerative diseases. It was previously acknowledged as the top ten causes of death in the USA [2], and its mortality and morbidity rate in the elderly has only risen substantially since then [3]. Neurodegeneration in AD is caused by the abnormal processing and polymerization of normally soluble proteins [4]. The general consensus regarding the pathogenesis of AD involves the progressive extracellular aggregation of amyloid-beta (Aβ) plaques as well as intracellular aggregation of neurofibrillary tangles (NFTs) composed of hyperphosphorylated tau proteins in the cortex and hippocampus [5]. More specifically, amyloid pathogenesis initially begins with dysfunctional cleavage of the amyloid precursor protein (APP), producing insoluble Aβ fibrils. Consequently, these Aβ fibrils then polymerise into insoluble amyloid fibrils that eventually aggregates into plaques. This polymerisation further causes the activation of kinases, resulting in hyperphosphorylation of tau proteins, and the eventual aggregation of insoluble NFTs. The progressive deposition of Aβ plaques and NFTs is followed by microglia recruitment surrounding the plaques, promoting the activation of microglia and local inflammatory response, leading to neurotoxicity [4]. Age, although not a direct cause, is the main risk factor associated with AD. The prevalence of AD increases significantly with age. An estimated 3% of adults aged 65–74, 17% of adults aged 75–84 and 32% adults aged 85 or older suffer from the disease [6]. Other potential risk factors such as chronic stress, genetic mutations and poor social relationships may also contribute to the pathogenesis of AD by facilitating Aβ deposition in the hippocampus [7, 8].

AD is a progressive disease where early symptoms of memory deterioration later develop into imparied social behavior and motor movements in patients. Patients suffering from AD typically progress slowly through four stages: the preclinical, early, middle, and late stages, each with varying clinical symptoms. During the pre-clinical stage, the individual experiences mild memory loss and early structural changes to the hippocampus, with no functional impairment of their daily activities [9]. Patients in the early stages experience progressive loss of concentration and memory and start to display depressive-like behaviors [10]. Should the disease progress to the moderate stage, this results in worsened memory and concentration, with difficulty in performing daily actions. Finally, patients suffering from late-stage AD will lose the ability to recognize individuals while also gradually losing control of their motor functions [5]. Currently, there are no known methods to prevent progression of Alzheimer’s, with the only clinical treatment available being drugs to alleviate the symptoms [11]. However, patients taking these drugs may experience side effects, such as gastrointestinal, fatigue, and potential abnormalities in breathing [12]. Hence, there is a need to develop a non-pharmacological presroach to prevent or delay the onset of AD.

The environmental enrichment paradigm was first explored by Donald Hebb, who demonstrated that socially enriched rats performed better in problem solving tests compared to their counterparts in standard housing [13]. Following this, Rosenzweig further established the concept of EE, whereby he classified EE as the combination of social stimulation along with the presence of various inanimate objects [14]. In the modern laboratory setting, EE is defined as the improvement of animal care quality in captive animals via the provision of appropriate stimuli needed to enhance physiological and psychological well-being [15]. For lab rodents, an enriched environment typically consists of larger cages and the presence of interactable objects as well as the rodents being housed together to stimulate social interactions [16]. Previously, studies have found that housing lab animals in EE had beneficial effects on their brain structure and behaviors. The main findings regarding the effects of EE on the brain can be summarized as improved spatial learning and memory consolidation [17, 18, 19], reduction of behaviors associated with depression and anxiety [20, 21, 22, 23], and reduction in age-related memory impairment [24, 25]. Initial studies investigating the effects of EE in preclinical models of neurodegenerative diseases were conducted in the early 2000s, particularly in Huntington’s disease and AD. In this regard, van Dellen et al., Hockly et al., and Spires et al. were some of the first to investigate the effects of EE on Huntington’s disease [26, 27, 28]. This led to several studies investigating the effects of EE on preclinical AD models, with varying degrees of success [29, 30]. Regardless, these initial studies were essential as they laid the foundation for EE as a potential therapy to combat neurodegenerative diseases. Throughout the past decade, many reviews have highlighted the effectiveness of EE against AD pathogenesis [31, 32, 33, 34, 35, 36]. Most notably, it has been shown that EE reversed Aβ pathology as well as reduced senile plaque aggregation in transgenic AD mice [37, 38]. Despite this, there is a lack of understanding regarding the molecular mechanisms associated with these changes in the brain. As such, this review serves not only to give an update to pre-existing literature but also to compile potential molecular pathways associated with EE and how it could influence the pathogenesis of AD as well as improve structures of the brain affected by it.

## Environmental Enrichment as a Neuroprotective Strategy in the Hippocampus

Studies regarding AD have mainly focused on the entorhinal cortex (EC) and hippocampus due to the disease’s effect on memory formation, spatial navigation, and motor behavior [39]. Furthermore, the neuronal dysfunction of the EC-hippocampal network has also been previously implicated during the early stages of AD [40].

Traditionally, morphological and structural changes in the hippocampus, such as progressive loss of hippocampal volume, were observed in patients suffering from AD [41, 42]. The reduction of neurogenesis throughout the progressive stages has also been recorded [43, 44]. In this regard, several animal studies have previously highlighted the beneficial impacts of EE on hippocampal structures. It has been reported that hippocampal neurogenesis was promoted following exposure to EE [45]. EE primarily caused an increase in the proliferation of progenitor cells whilst also promoting cell survival in the hippocampus [46, 47, 48]. Similarly, this could also be observed in transgenic rodent models of AD, as EE restored impaired adult hippocampal neurogenesis after deposition of Aβ plaques [49, 50, 51]. The extent of neurogenesis varies between these studies, presumably due to differences in parameters such as the age of the rodents, duration of EE exposure, and differences in EE protocols used*.*

Additionally, both CA1 and dentate gyrus (DG) volumes in the hippocampus were also significantly increased after long-term exposure to EE, resulting in improved cognitive performance [52]. The increase in hippocampal volume could be attributed to the cumulative effects of EE, such as increased cell proliferation and dendritic arborization, as well as enhanced vascularity and dendritic complexity [53]. The effects of EE on hippocampal activity have also been of interest in recent years. Hippocampal activity is directly tied to the long-term potentiation (LTP) generated between hippocampal excitatory neurons, which are involved in the process of learning and the formation of memory [54]. In addition, synaptic dysfunction of the hippocampus is also implicated during the early stages of AD, leading to the progressive impairment of memories [55]. In recent AD studies using different transgenic rodent models, electrophysiological recordings of hippocampal LTP showed a marked decline in magnitude, suggesting reduced synaptic plasticity of the hippocampus [56, 57, 58]. Synapse loss and dysfunction had also been heavily correlated with cognitive decline associated with AD subjects [59, 60].

The effects of EE on synaptic plasticity in the hippocampus have been frequently discussed in multiple rodent models. Hippocampal LTP was found to be enhanced following exposure to short-term EE [61, 62]. Whole-brain deep sequencing analysis post-EE exposure revealed upregulation of genes associated with synaptic plasticity, such as *brain-derived neurotrophic factor (BDNF)* and the *N*-methyl D-Aspartate receptor subtype 2B (*GRIN2B)* genes [52]. In seizure-prone models of rodents, EE preserved hippocampal LTP in CA1 neurons, while preventing loss of synaptic density and dendrite branching [63]. A recent study also investigated the effects of EE on hippocampal plasticity in permanent middle cerebral artery occlusion models of mice. After 28 days of exposure to EE, protein expression for synaptic proteins (such as growth-associated protein 43 (GAP-43), synaptophysin, and postsynaptic density protein 95 (PSD-95)) were found to be significantly increased compared to the mice housed in standard housing conditions, leading to the formation of higher amounts of hippocampal synapses [64]. Moreover, these effects could also be observed in transgenic AD rodents. A prime example can be found in the use of EE to enhance synaptic plasticity in transgenic AD mice expressing mutations in the amyloid precursor proteins (APP) and presenilin 1 (PS1) genes (APPswe/PS1**Δ**E9). Furthermore, 4 weeks of EE prevented synaptic impairment induced by Aβ Oligomer deposition [65], indicating the potential of EE as both a neuroprotection and treatment strategy. A summary of the effects of EE on the hippocampus is tabulated in Tables 1 and 2.

**Table 1:** Studies investigating the effects of environmental enrichment on the hippocampus in non-neurodegenerative models of rodents

Rodent species	Gender	Age of enrichment	Duration of enrichment	Housing conditions	Findings	Reference
Mouse (C57B16/J)	Female	P28 (prepubertal)	11 months	Running wheels, tunnels, houses, toys *N* = 7 per cage	• ↑ CA1 and dentate gyrus volume • ↑ dentate gyrus neurons • ↑ genes associated with synaptic plasticity and transcription regulation	[52]
Rat (Wistar)	Male	P21 (Prepubertal)	40 days	Tunnels, cylinders, metal/plastic objects, running wheel, nesting materials *N* = 10 per cage	• ↑ volume in the ventral hippocampus and the medial prefrontal cortex	[276]
Rat (Sprague–Dawley)	Male	21 months (aged)	3 weeks	Toys, cylinders, tunnels, metal/plastic objects *N* = 3 per cage	• ↑ synaptic plasticity through induction of LTP and long-term depression (LTD)	[61]
Rat (Sprague–Dawley)	Male	P28–30 (prepubertal)	3–5 months	Tunnels, ladders, boxes, plastic toys *N* = 4 per cage	• No changes in LTP in the CA1 region and dentate gyrus • ↓ LTD, ↓ depotentiation and altered paired-pulse inhibition of the population spike	[272]
Mouse (C57BL/6JRj)	Female	5 weeks (pubertal)	6 months	Toys, tunnels, and hideouts *N* = 40 per group	• ↑ hippocampal neurogenesis • ↑ synaptic plasticity through upregulation of genes involved in neuronal plasticity	[273]
Rat (Sprague–Dawley)	Male	P21 (prepubertal)	13 weeks	Aspen wood bedding, 3 running wheels, climbing ladder, slide tunnel, 6 mazes *N* = 12 per group	• ↑ hippocampal neurogenesis • ↑ cortical thickness • ↑ expression of genes involved in brain plasticity	[270]
Rat (Wistar)	Male	1.5–2 months (adult)	10 days	Toys, ladders, and tunnels *N* = 10–12 per group	• ↑ synaptic plasticity through induction of LTP	[277]
Mouse (C57B16/N)	Female	6–8 weeks old (pubertal and adult)	4 weeks	Toys, houses, maze-like tube system *N* = 10 per cage	• ↑ hippocampal neurogenesis	[45]
Mouse (BalbC)	Female	6 months old (adult)	45 days	Tunnels, running wheel, pieces of wood, nesting materials, small plastic houses with stairs *N* = 10 per cage	• ↑ hippocampal neurogenesis • ↑ synaptic plasticity through increased dendritic spines	[46]
Mouse (Fgfr1^T2A−H2B−GFP^ and Fgfr2^T2A−H2B−mCherry^)	Male & female	2 months old (adult)	10/14 days	Two running wheels, three tunnels, water and food station, standard bedding, bedding squares *N* = 10 per cage	• ↑ hippocampal neurogenesis	[48]

**Table 2:** Recent studies investigating the effects of environmental enrichment on the hippocampus in neurodegenerative models of rodents

Rodent species	Gender	Age of enrichment	Duration of enrichment	Cage conditions	Findings	References
Mouse (APOE4 transgenic)	Male	P21 (prepubertal)	24 weeks	Running wheel, labyrinth, bedding, ladder, house, chains, wooden blocks *N* = 5 per cage	• ↓ hippocampal neurogenesis • ↑ hippocampal apoptosis	[250]
Mouse (3xTg-AD)	Male	P21 (prepubertal)	6 months	Running wheel only *N* = 4 per group	• ↑ hippocampal neurogenesis	[49]
				Tunnels, climbing materials, tilted running wheels *N* = 4 per group		
Mouse (TgCRND8)	Female	P30 (prepubertal)	120 days	Gnawing wood, sisal rope, tunnels, balls, soft materials, wooden ramps, ladders, plastic stairs, running wheels *N* = 3–4 per cage	• ↑ cell proliferation and hippocampal neurogenesis • ↑ structural plasticity proteins (synaptophysin, ARC and GAP43)	[278]
Mouse (APP_Sw,Ind_)	Female	4 months (adult)	7 weeks	Plastic play tubes, 2–4 hideouts, running wheel *N* = 6–8 per cage	• ↑ hippocampal neurogenesis • ↑ number, dendritic length and projection to CA3 region of mature neurons	[50]
Mouse (APP_SWE_/PS1_dE9_)	Male	6 months (adult)	6 months	Wooden and plastic blocks, platforms, ball, running wheel, mouse hut *N* = 4–5 per cage	• ↑ synaptic density in CA1 region	[75]
Mouse (APPswe/PS1ΔE9)	Male	P21 (prepubertal stage)	1 month	Running wheels, tunnels, toys *N* = 3–4 per cage	• ↑ cell proliferation in the dentate gyrus • ↑ survival of new neurons • ↑ hippocampal LTP • ↑ astrogenesis	[66]
Mouse (PDAPP-J20)	Female	5 months (adult)	3 months	Toys, extra nesting materials, houses, tubes, no running wheels *N* = 5 per cage	• Prevention of astroglial morphological changes induced by Aβ	[279]

Besides influencing the structure and neuronal activity of the hippocampus, EE also functions as a neuroprotective and treatment strategy by preventing and/or reducing the accumulation of Aβ plaques during the progressive stages of AD. Early exposure to EE can attenuate amyloid pathology as evidenced by decreased Aβ deposition in the cortex and hippocampus of transgenic mice with overproduction of Aβ peptides and accelerated amyloid deposition (FAD-linked APPswe/PS1ΔE9) [37]. Additional studies also reported significantly reduced Aβ levels in the hippocampus and cortex post-EE exposure, before the onset of amyloid deposition [66, 67]. In tandem, tau phosphorylation was also significantly decreased, resulting in reduced formation of NFT and neurophil threads [68]. The preventive effects of EE on amyloid deposition could be attributed to increased amyloid clearance, as EE induced expression of Aβ degrading molecules, altered Aβ transporters levels as well as activated microglial clearance [69, 70]. Interestingly, an earlier study highlighted an increase in Aβ deposition and levels resulting from early exposure to EE [71, 72]. Furthermore, some studies have even recorded no changes in Aβ deposition, though they noted an improvement in cognitive functions [73, 74]. Presumably, the differences in results could be due to variances in parameters, such as the model of transgenic rodents used, housing conditions for EE, and sex of rodents.

Although the neuroprotective property of EE is apparent during early intervention, its effectiveness in mid-life and aged models of AD has only recently gained attention. EE exposure during mid and late-life phases has yielded conflicting results in recent studies. Initially, a study conducted by Stuart et al. only observed an increase in CA1 synaptic density after long-term EE exposure to 6 months old mice, with no noticeable changes to Aβ plaque load in the hippocampus and neocortex [75]. Additionally, long-term stimulation of EE did not affect Aβ oligomer levels but was found to have a profound effect on hippocampal senile plaque concentration in mid-life mice (8–12 months) [38]. In contrast, Fulopova et al. described regional changes in amyloid deposition, with Aβ plaque formation in the somatosensory and primary motor cortex being significantly reduced while observing no changes in the prefrontal cortex of mid-life APP/PS1 mice [76]. Similarly, in aged APPsw transgenic mice (20–22 months), 4 months of EE exposure did not alter Aβ deposition but instead improved cognition in these mice [74]. On the other hand, Mainardi et al. observed a decrease in hippocampal Aβ oligomer levels in 17-month-old mice, attributing this to the increased synthesis of the Aβ-degrading enzyme neprilysin caused by EE [77]. Although the efficacy of EE during the early stages has been well documented, these findings suggest that more studies need to be conducted to determine its effectiveness during mid-late life.

## Potential Molecular Mechanisms Associated with EE in the brain

Despite existing evidence supporting the effectiveness of EE as a non-pharmacological approach in delaying the onset of AD, the underlying mechanisms behind how it achieves this has yet to be well understood. EE stimulates growth and proliferation pathways which delay the progression of AD. As such, this subtopic will discuss the molecular mechanisms associated with counteracting the effects of AD.

A major point of interest in EE is in its effect on hippocampal neurogenesis. As previously mentioned, EE serves as a neuroprotective strategy by promoting neurogenesis, as indicated by increased cell proliferation and cell survival in the hippocampus [46, 47, 48]. A plethora of studies indicate that EE promotes the expression of neurotrophins, such as brain-derived neurotrophic factor (BDNF) [78, 79, 80, 81]. and nerve growth factors (NGF) in the hippocampus [82, 83]. BDNF is a neurotrophin that induces the differentiation and survival of neurons [84]. BDNF also activates downstream pathways associated with regulating excitatory and inhibitory synaptic transmission in the adult brain. These actions are primarily exerted via BDNF activation of multiple downstream pathways upon binding with its receptor, tyrosine receptor kinase B (TrkB) [85].

### Phosphoinositide-3 Kinase/Protein Kinase B Pathway

**Fig. 1:**
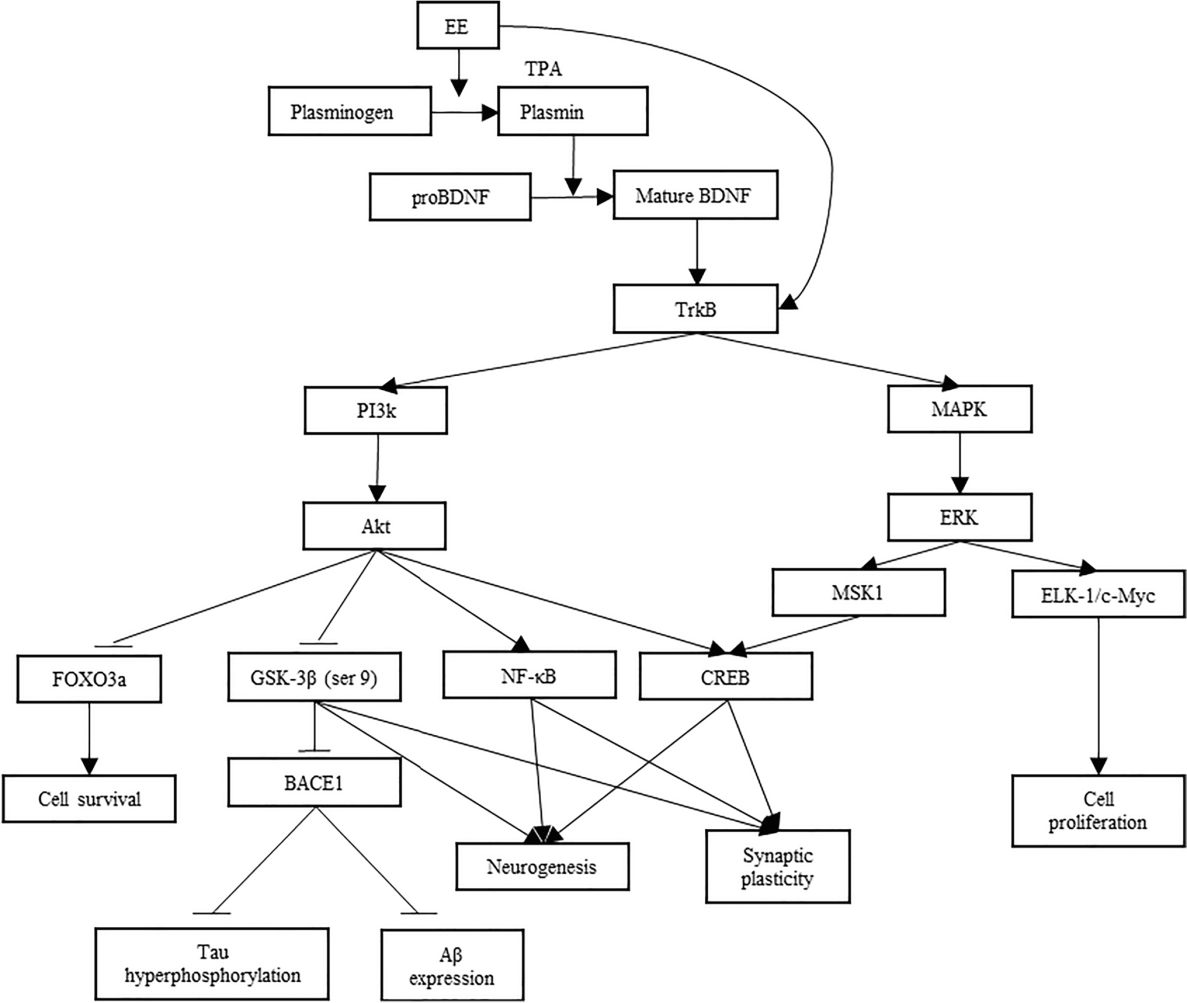
Environmental enrichment (EE) causes upregulation and activation of tissue plasminogen activator which converts plasminogen into plasmin. The increase in plasmin facilitates the conversion of inactive proBDNF to active mature BDNF. EE also upregulates expression of tyrosine kinase b (TrkB) in conjunction with the increase in mature BDNF. Binding of mature BDNF to TrkB causes activation of the multiple pathways, mainly the phosphoinositide-3 kinase/protein kinase B (PI3k-Akt) and the mitogen-activated protein kinases/extracellular signal-regulated kinase (MAPK/ERK) pathways. Activation of the PI3k-Akt leads to multiple downstream effects. Phosphorylated-Akt (p-Akt) inactivates FOXO3a protein, leading to cell survival. Besides that, p-Akt also phosphorylates glycogen synthase kinase 3 beta (GSK-3β) at serine 9 (ser 9), inactivating it. This causes the inhibition of BACE1 expression, which reduces expression of amyloid beta plaques and decreases tau hyperphosphorylation. Furthermore, inhibition of the GSK-3β pathway also promotes neurogenesis and synaptic plasticity. p-Akt also causes activation of NF-κB protein complex and CREB protein, leading to increased neurogenesis and enhanced synaptic plasticity. BDNF binding also causes the activation of the MAPK/ERK/MSK pathway, which also regulates neurogenesis and synaptic plasticity via the phosphorylation and activation of the CREB pathway. This pathway could also potentially promote cell proliferation via regulation of ELK-1 and c-Myc genes

The initial increase in BDNF levels induced by EE could be due to upregulated expression of tissue plasminogen activator (tPA), which converts plasminogen into plasmin and consequently increases conversion of proBDNF (inactive) into mature BDNF [86, 87]. The mature form of BDNF then preferentially binds to TrkB instead of neurotrophin receptor p75 (p75^NTR^). Concomitantly, the expression of TrkB receptors is also increased in response to EE [22, 87, 88]. The binding of mature BDNF to TrkB subsequently causes activation of multiple pathways (Fig. 1). One of them is through the activation of the phosphoinositide-3 kinase/protein kinase B (PI3k-Akt) pathway as evidenced by the increased phosphorylation of Akt after different periods of EE exposure [46, 87, 89].

### Nuclear Factor-Kappa B Pathway

Although the *nuclear factor-kappa B* (NF-κB) pathway has previously been extensively involved in the development and progression of cancer, recent reviews have highlighted the potential of the NF-κB pathway as a therapeutic target for AD [90, 91]. The NF-κB belongs to a family of transcription factors typically associated with the regulation of inflammation. In AD, the NF-κB is essential for the modulation of beta-secretase 1 encoded by the BACE1 gene. BACE1 initiates the production of Aβ with gamma-secretase, which cleaves a portion of the amyloid precursor proteins (APP), resulting in the accumulation of Aβ plaques between hippocampal neurons [92]. Overactivation of the NF-κB pathway leads to increased promoter activity of APP and BACE1 genes, resulting in elevated Aβ accumulation [93, 94]. Moreover, NF-κB codes for various target genes relevant to the formation of long-term memory and hippocampal plasticity [95], indicating that dysregulation of this pathway could lead to impairments in hippocampal function. P-Akt regulates the transcriptional activity of NFκB by inducing the phosphorylation and consequent degradation of inhibitor of κB (IkK), thereby activating it [96].

Studies regarding the NF-κB pathway yielded conflicting results. For example, EE-dependent activation of NFκB promoted the expression of hippocampal genes essential for learning plasticity and learning (e.g., BDNF and CamK2D) in old age rats, resulting in improved adult neurogenesis and healthier synaptic densities [97]. In addition, early-life EE exposure in an accelerated aging model of mice (SAMP8 mice) causes downregulation of inflammatory genes associated with NFκB (IL-6, Cxcl10) while also upregulating antioxidant genes (Hmox1, Aox1, Cox2) [98]. In contrast, exposure of mid-life 5xFAD mice (mice expressing human APP and PSEN1 transgenes with five AD-linked mutations) to 3 months of EE caused an upregulation of microRNA-146a, resulting in down-regulation of NF-κB and inhibition of astrocytic inflammation [99]. Reduced expression of NF-κB was also seen in EE-exposed diabetic streptozotocin rats, consequently leading to decreased hippocampal neuronal loss and astroglial inflammation whilst also increasing synaptic density [100]. Additionally, EE also influences the expression of the BACE1 gene. Though not in AD models, EE reduced BACE1 expression in both young and aged mice following chronic variable stress [101]. As such, the NF-κB is a challenging pathway to focus on since it regulates the transcription of both Aβ related genes and genes related to hippocampal function. Although EE regulates this pathway bidirectionally, its corresponding effects are different. On one hand, upregulation of NF-κB is essential for preserving and improving hippocampal structure. On the other hand, increasing NF-κB signaling could also lead to increased BACE1 expression, leading to increased Aβ deposition [93]. Inversely, downregulation of NF-κB could lead to a reduction in BACE1 expression [102]; however, it could also have profound effects on other genes involved in cell survival, cell proliferation, and cell differentiation. The NF-κB pathway is activated during aging and contributes to the pathogenesis of age-related diseases. Hence, EE could potentially aid in the modulation of this pathway by preventing over-activation during the aging process.

### Glycogen Synthase Kinase 3 Beta Pathway

Another pathway that could potentially be influenced by EE is the glycogen synthase kinase 3 beta (GSK-3β) pathway. Dysregulation of GSK-3β is indeed associated with increased deposition of Aβ plaques in the hippocampus. Furthermore, the overactivation of GSK-3β promotes hyperphosphorylation of toxic tau protein and consequently the formation of NFT [103]. GSK-3β overexpression also causes morphological alterations in hippocampal granule neurons like that of AD patients [51]. In contrast, inhibition of GSK-3β has been shown to attenuate cell death associated with the early introduction of neurotoxic Aβ peptides, and ameliorate behavioral changes induced by AD [104, 105]. The phosphorylated state of Akt (p-Akt) inhibits GSK-3β activity by phosphorylation at the Ser9 site [106], potentially causing reduced tau hyperphosphorylation and reversing synaptic abnormalities through inhibition of BACE1 [51, 107]. Indeed, this may be one of the pathways associated with the decreased levels of Aβ after exposure to EE [77]. Furthermore, inhibition of GSK-3β can enhance adult neurogenesis. Previous studies have demonstrated that the inhibition of this kinase led to increased neural stem cell proliferation as well as increased neuronal differentiation in both in vivo and in vitro models [108, 109]. Similarly, EE restored proliferation of neural precursor cells in GSK-3β knock-in mice [110] However, its effects were only noticeable in males, but not in their female counterparts.

Overactivation of GSK-3β was also rescued by EE exposure. Specifically, EE reversed morphological alterations induced by GSK-3β overactivation in adult neurons, resulting in improved synaptic plasticity [51]. However, the upstream signaling cascades involved in this particular process remain poorly understood. Realistically, a combination of different pathways could lead to such attenuation as both the PI3k-Akt and NF-κB signaling cascades are involved in regulating GSK-3β activity [107, 111].

### Cyclic-AMP Response Element-Binding Pathway and Forkhead Box O3 Transcription Factor

The activation of the PI3k-Akt pathway also promotes adult neurogenesis via the involvement of different downstream signaling cascades. These pathways have mainly been associated with cell proliferation, cell survival, and cell differentiation. In addition to the NFκB and GSK-3β pathways, the cyclic-AMP response element-binding (CREB) protein has been shown to play an essential role in hippocampal function as it mediates the expression of genes involved in cell proliferation and survival [112, 113]. Activation of CREB via phosphorylation of PI3k-Akt has previously been shown to trigger the proliferation of adult hippocampal progenitor cells [114]. Furthermore, activation of CREB led to enhanced hippocampal synaptic plasticity as well as heightened neuronal excitability [115, 116, 117]. The majority of the studies conducted have indicated that EE increased expression and/or phosphorylation of CREB in some capacity [87, 116, 118]. However, one study failed to replicate this effect [119]. This could be due to a lack of physical enrichment, as the later study lacked the novelties needed to stimulate physical activity. Indeed, physical activity has been shown to elicit activation of CREB in the hippocampus [120, 121]. Besides that, PI3k-Akt can enhance cell survival via the involvement of the Forkhead box O3 transcription factor (FOXO3a). FOXO3a is primarily involved in the process of apoptosis via upregulation of genes essential for cell death, such as Bim and p53 [122]. Phosphorylation of FOXO3a by Akt causes its localization in the cytosol as an inactive complex bound [123]. Inactivation of FOXO3a prevents apoptosis of cells, leading to increased cell survival.

### Mitogen-Activated Protein Kinases/Extracellular Signal-Regulated Kinase Pathway and Other Potential Factors

Alternatively, binding of BDNF to TrkB could also activate the Mitogen-activated protein kinases/extracellular signal-regulated kinase (MAPK/ERK) pathway, which is essential in cell proliferation, differentiation, migration, and apoptosis [124]. Similar to the PI3k/Akt pathway, MAPK/ERK promotes cell proliferation through activation of CREB [125]. Activation of CREB through the MAPK/ERK pathway is mediated by pp90 ribosomal S6 kinase (RSK), mitogen- and stress-activated protein kinase (MSK)1/2. MAPK/ERK could also influence cell proliferation via other downstream transcription factors, such as ELK-1 and c-Myc [126, 127]. Previous studies have reported how EE influences the MAPK/ERK pathway. Bengoetxea et al. reported that animals reared in EE reversed neuronal and vascular deficiencies induced by the effects of vandetanib, a Trk inhibitor [89]. The group attributed this recovery to the activation of PI3k-Akt and MAPK pathways caused by EE-mediated BDNF-TrkB binding. Furthermore, another group noted that MSK1 is an essential component for EE-induced effects. More specifically, they noted that EE had diminished spinogenesis and SGZ progenitor proliferation in MSK1 knockout mice compared to their wild-type counterparts [128]. In contrast, other studies have recorded no changes in hippocampal ERK levels after exposure to EE [87, 129].

### Wingless-Type MMTV Integration Site Family/β-Catenin Signaling Pathway

Interestingly, one of the least discussed pathways regarding AD is the *wingless-type MMTV integration site family* (Wnt)/β-catenin pathway. This pathway was first recognized for its involvement in carcinogenesis [130]. However, a growing amount of evidence has supported its involvement in the pathology of AD [131, 132]. The Wnt/β-catenin pathway is responsible for a number of essential biological processes, such as cell survival and cell proliferation, as well as synaptic plasticity [133]. Inhibition of the Wnt signaling cascade is often studied to prevent proliferation and migration in cancer cells [134, 135, 136]. Moreover, inhibition of the Wnt causes synthesis of Aβ oligomers and aggregation of Aβ plaques [137, 138]. Restoration of this dysregulated pathway could ameliorate symptoms and delay the progress of AD. Although limited, recent studies have focused on how EE affects the Wnt signaling cascade. Surprisingly, EE induced activation of Wnt/β-Catenin in rodents with vascular dementia, effectively restoring spatial learning and memory [17]. Moreover, exposure of middle-aged and aged rats to exercise and enrichment induced hippocampal activation of the canonical Wnt pathway [139, 140], potentially improving hippocampal plasticity and neuronal densities. Although these are promising effects, more studies are needed to determine how EE with and/or without the presence of exercise could affect the Wnt signaling cascade.

### Nerve Growth Factor (NGF), pro-NGF, and the c-Jun N-terminal Kinase (JNK) Signaling Pathways

Although EE primarily produces their neuroprotective effects through BDNF, the signaling pathways induced by other neurotrophins should also be considered. Another promising area of interest is the basal forebrain due to its involvement in AD. The basal forebrain provides a major source of cholinergic innvervations to the hippocampus and cortical areas [141]. The loss of these cholinergic neurons could impair hippocampal processing information and potentially contribute to AD-related psychiatric symptoms [142]. Furthermore, a recent study also demonstrated that the neurodegeneration of basal forebrain precedes even that of the entorhinal cortex during the pathogenesis of AD [143]. Nerve growth factor (NGF) is essential for the functioning of the basal forebrain as it contributes to the formation of dendritic trees and modulates the activity of cholinergic systems projecting to the hippocampus [144]. During the pre-clinical and early stages of AD, NGF metabolism is dysregulated [145], leading to higher amounts of proNGF (precursor) which results in loss of cortical synapses and atrophy of cholinergic neurons in the basal forebrain. As such, current-day studies have focused on the biodelivery of NGF to degenerated cell bodies in the basal forebrain as a treatment method [146, 147, 148], in an attempt to resuscitate cholinergic signaling in the cortex and hippocampus [149].

**Fig. 2:**
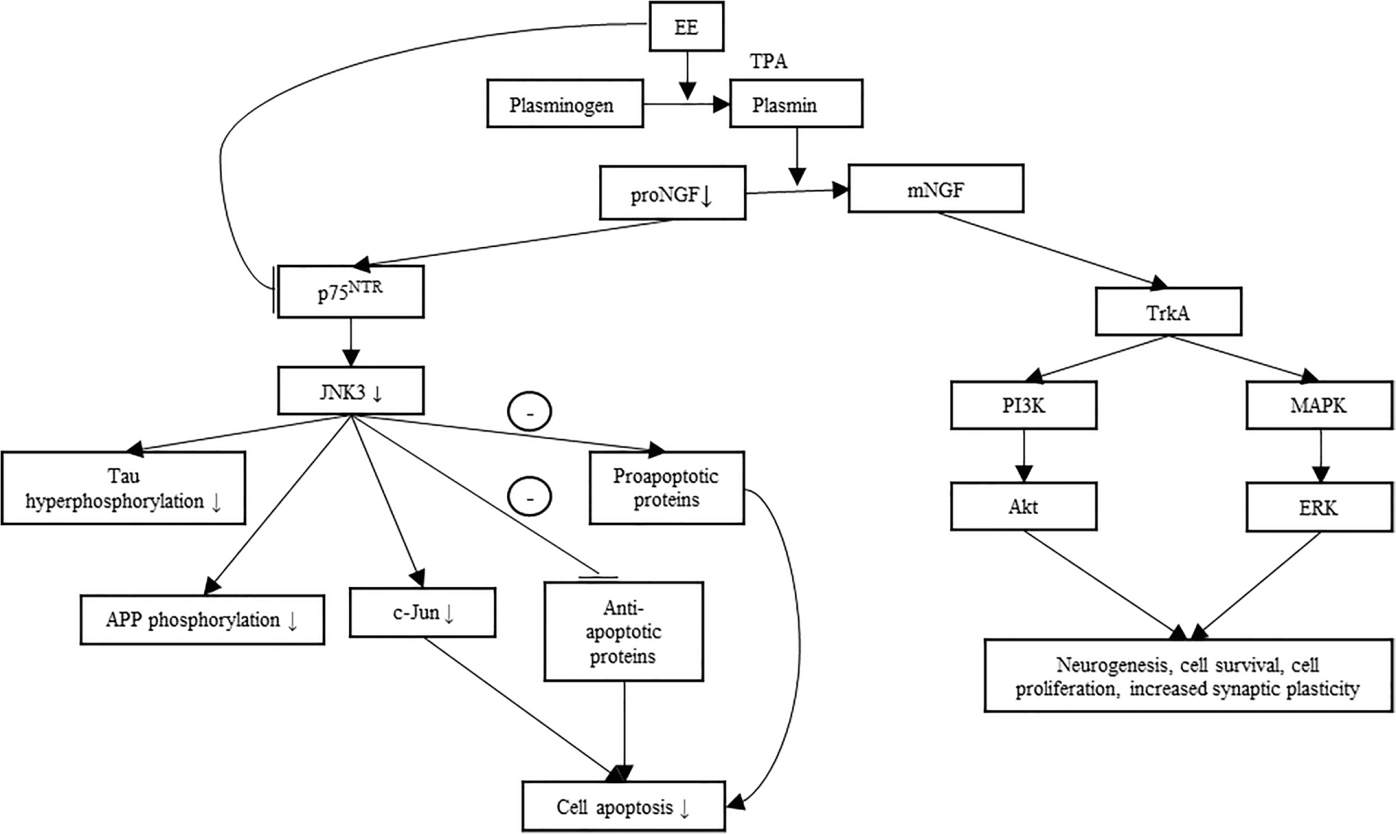
EE promotes the conversion of proNGF to mature NGF (mNGF) through increased plasmin levels. mNGF then binds to tyrosine kinase A (TrkA), which subsequently activates the PI3k-Akt and MAPK/ERK pathway and promotes neurogenesis, cell survival, cell proliferation and increased synaptic plasticity in the basal forebrain. As a result of this conversion, EE also indirectly decreases the activation of the JNK pathway signaling pathway by indirectly reducing proNGF levels (↓) and directly reducing p75^NTR^ levels (↓) in the brain. This results in lower JNK phosphorylation, consequently leading to decreased tau hyperphosphorylation, decreased APP phosphorylation and decreased cell death in the basal forebrain

The effects of EE on NGF are similar to its effects on BDNF expression, where multiple studies have noted an increase in NGF in response to both short and long-term exposure of EE [82, 150, 151, 152, 153]. Presumably, this increase in NGF levels could be attributed to the upregulation of plasmin, as plasmin functions to convert proNGF to mature NGF (mNGF). NGF’s mechanism of action is also similar to that of BDNF. However, one key difference is that NGF initially binds to tyrosine kinase A instead of tyrosine kinase B. This leads to the activation of the PI3k-Akt and MAPK-ERK pathways and consequently improves cell survival, neurogenesis and synaptic plasticity. However, what is more interesting and has been less discussed upon is how EE could potentially lead to decreased levels of its precursor protein, proNGF. Unlike BDNF, both proNGF and NGF are biologically active [154]. Although proNGF could bind to both TrkA and p75^NTR^, proNGF has a weaker binding affinity to TrkA compared to p75^NTR^ [155]. Under normal physiological conditions, proNGF is neurotrophic as it could activate TrkA downstream pathways [156]. However, that is not the case in AD patients. As previously mentioned, the conversion of proNGF to mNGF is dysregulated during AD, leading to decreased mNGF production and increased mNGF clearance [157, 158]. Furthermore, it has also been shown that in AD patients, TrkA receptor levels are reduced, with no changes in p75^NTR^ levels being observed [159, 160]. As a result, the increased concentrations of proNGF preferentially bind to p75^NTR^ instead of TrkA, inducing neurodegeneration in the basal forebrain via the activation of the apoptotic c-Jun N-terminal kinase (JNK) pathway (Fig. 2).

The JNK pathway has previously been shown to be implicated in the pathogenesis of AD [161, 162]. This is evidenced by early post-mortem and AD models showing increased JNK concentrations and activity [163, 164]. One of the potential activation signals of the JNK pathway occurs via binding of proNGF to p75^NTR^. However, in-vitro models have also reported instances of Aβ oligomers activating the JNK pathway [165, 166, 167], resulting in cell apoptosis and abnormal dendritic spine morphology.

Under AD pathological conditions, the increased concentrations of proNGF as well as Aβ oligomers bind to p75^NTR^, causing the phosphorylation of JNK3, the primary JNK isoform localized in neurons [168]. Activation of the JNK signaling pathway leads to contributes to several phenotypes of AD, and these include (i) increased hyperphosphorylation of tau proteins at serine 422, contributing to increased formation of NFT [169], (ii) increased Aβ peptide and plaque levels as JNK functions as a major kinase for APP phosphorylation at threonine 688 [170], and (iii) increased cell death due to activation of proapoptic proteins (BMF, BIM) [171, 172], inhibition of anti-apoptotic proteins (Bcl2 and Bcl2 homologs) [173, 174], and phosphorylation of c-Jun which mediates cell cycle progression and apoptosis [175, 176].

Although there is a lack of studies regarding the effects of EE on proNGF, conversion of proNGF to NGF could potentially reduce Aβ production, tau hyperphosphorylation, and cell death via downregulation of the JNK pathway. Inhibition of the JNK pathway has previously shown to be effective in delaying the progression of AD hallmarks [177, 178, 179], as well as prevent neuronal cell death induced by JNK pro-apoptotic signaling [180]. Recently, Cho and Kang reported a significant decrease in both proNGF and p75^NTR^ levels in a Parkinson’s Disease mice model following 4 weeks of EE, leading to neuroprotective effects on dopaminergic neurons [181]. However, the effects of EE on proNGF and p75^NTR^ in AD models have yet to be investigated and should become a topic of interest in future EE studies.

### Fibroblast growth factors (FGF)

Several reviews have been written regarding the mechanism of action of fibroblast growth factors [182] as well as their involvement in AD [183, 184]. Treatment of AD with different members of FGF have been shown to induce neuroprotective properties in different preclinical models of AD, especially in FGF2. For example, FGF2 has been shown to be a viable treatment method in preclinical models of AD. Chen et al. demonstrated that both low and high molecular weight FGF2 were able to induce neuroprotective effects on astrocytes against Aβ-induced cytotoxicity [185]. Additionally, another study has shown that FGF2 gene transfer is able to restore hippocampal function in a transgenic model of AD mouse, whilst also enhancing Aβ phagocytosis and reducing Aβ production in primary cultured microglia and neurons after FGF2 infection [186]. Furthermore, Katsuori et al. also noted similar results, where FGF2 treatment of APP_23_ transgenic mice restored spatial memory function, reduced Aβ levels and tau pathologies, and increased astrocytic survival in the dentate gyrus compared to control mice [187]. However, the effects of EE on FGF expression in AD models have been relatively neglected compared to its effects on other neurotrophins. Although not in AD models, both short- and long-term EE has previously been shown to upregulate hippocampal expression of FGF2 in different models of rodent [48, 188, 189]. Furthermore, EE could also promote adult hippocampal neurogenesis through activation of fibroblast growth factor receptors and fibroblast growth receptor substrates [48]. We hypothesize that EE could ameliorate hallmarks of AD by improving FGF levels in the brain. However, more studies have to be conducted in order to better understand the effects of EE on FGF levels in AD models.

Other potential factors which could contribute to the effects of EE include increased expression of neurotrophin-3 and insulin-like growth factor-1 [87], improvements of N-methyl-D-aspartate receptor histone acetylation [190], and changes in metabotropic glutamate receptor mGluRs [191, 192], consequently leading to downstream activation of other undiscussed pathways.

**Fig. 3:**
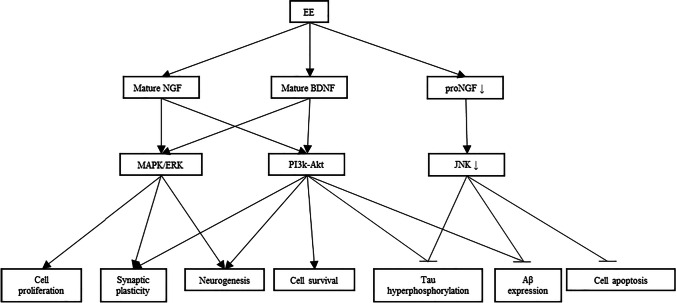
Simple summary of the effects of EE in AD. EE causes an increase in mature NGF and BDNF levels, leading to activation of the MAPK/ERK and PI3k-Akt pathways. Consequently, activation of these pathways leads to increased cell proliferation, increased synaptic plasticity, neurogenesis and increased cell survival in the brain. Activation of the PI3k-Akt pathway could also inhibit tau hyperphosphorylation and reduce Aβ expression levels in the brain. EE also indirectly downregulates the JNK pathway as it facilitates the conversion of proNGF to mature NGF. Downregulation of the JNK pathway mainly leads to reduced cell death in the brain, but could also reduce tau hyperphosphorylation and decrease Aβ expression levels

As such, the effects of EE are not confined to a single pathway but rather the activation of multiple downstream pathways working in tandem to produce these effects. To summarize, EE not only delays the progression of AD by affecting its pathogenesis, but also counteracts the neurodegeneration of AD by activating cell regenerative pathways (Fig. 3). As current studies are far and few in between, further investigations in this field hold the promise of more discoveries to be made.

## Behavioral Effects of EE and Its Potential Application in Humans

Alongside the progressive decay in cognitive and motor functions, patients with AD also experience gradual alterations in their behavioral patterns. Most notably, they tend to experience behavioral and psychological abnormalities such as psychosis (in the form of hallucinations and delusions), aggressive-like behavior, and depression and anxiety [193]. Normal individuals who experience healthy aging are at no greater risk of developing depression compared to younger adults [194]. In contrast, patients with AD are more likely to display depressive symptoms compared to their healthy counterparts [195]. It is suggested that the stage of AD may influence the risk of developing depression, however results remain inconclusive.

Fortunately, the effects of EE on social behavior have been well documented throughout the past decade. Previous studies have suggested the use of EE in ameliorating anxiety and depressive-like behaviors. Implementation of EE during the early life phase has demonstrated anxiolytic and antidepressant effects on depressed rodents [23, 196]. Moreover, the effects of EE have also been demonstrated in middle and late-life rats [197, 198], implying its effectiveness throughout different stages of life. Additionally, rats exposed to EE displayed reduced anxiety-like behaviors, as seen by increased exploratory behavior during the elevated plus maze and open field tests [23, 199]. This was also seen in a triple transgenic mouse model of AD [200], indicating that EE can also ameliorate AD-induced behavioral alterations.

Although how EE exhibits these antidepressant and anxiolytic properties remains relatively unknown, it is postulated that it could be a result of EE-induced hippocampal neurogenesis [201]. Furthermore, the antidepressant properties of EE could also be a result of the upregulation of neurotrophic factors, such as BDNF and vascular endothelial growth factors, which are responsible in regulating neurogenesis and angiogenesis [20, 202]. Nevertheless, the possibility that EE could also exert these effects via different areas of the brain should not be dismissed. For example, it has been suggested that the anxiolytic effects of EE is associated with down-regulation of amygdalar corticotropin-releasing factor receptor 1 expression [203]. In contrast, another study has claimed that the anxiolytic effects of EE could be modulated by serotonin neurons located in the dorsal raphe nucleus [204].

Recently, an increasing amount of evidence has hinted that EE can improve memory and delay age-related cognitive dysfunctions [205, 206]. Early exposure of EE to AD transgenic rodents was previously found to delay the onset of memory deficits as well as improve neuropathological hallmarks [207, 208, 209]. Moreover, late-life EE was also found to be an effective strategy in improving learning and preserving memory in aged AD transgenic rodents [97, 210]. As the process of learning and memory retention is a primary function of the hippocampus, the increase in hippocampal neurogenesis and improvements in synaptic plasticity could be a key benefit of EE [50, 206]. Furthermore, epigenetic alterations induced by EE, such as modifications to histone acetylation and DNA-methylation, are postulated to lead to a relaxed chromatin structure, improving the expression of genes and proteins essential for learning and memory [97, 208, 211].

While clinical studies are lacking at present, looking at each aspect of EE separately provides us a greater insight into their potential application when it comes to humans. Fundamentally, EE can be broken down into the presence of physical activity, social interaction, and cognitive stimulation [212]. The effects of exercise in the treatment of behavioral disorders is the most well-researched aspect of EE. Based on several meta-analyses and reviews, exposure to physical activities can be an effective method of treatment for depression in adults, rivaling the effects of antidepressant drug therapy [213, 214]. Moreover, treatment of late-life depression in elderly individuals with aerobic exercise seems to also improve depressive-like symptoms [215, 216], with the added benefit of avoiding the side effects of antidepressant medications. Likewise, patients with diagnosed AD who are under long-term exercise regimes had improved cognitive functions [217], likely due to improved vascular blood flow, hippocampal volume, and increased neurogenesis [218, 219]. In addition, recent meta-analyses have also indicated that EE could also benefit memory preservation in older individuals. A meta-analysis comprising of 28 studies (2156 participants) conducted by Zhidong and his team found that physical exercise could improve working memory in older individuals [220]. Aghjayan et al. also conducted a meta-analysis investigating the effects of physical activities in older adults and found that aerobic exercises positively affect episodic memory as well [221]. Similarly, the same effects can also be observed in elderly patients with AD, with Jia et al. reporting that physical activities contribute to their improvement in cognitive functions [222].

Subsequently, the presence and quality of social interactions are also essential for maintaining a healthy state of mind. Accumulating evidence has suggested that social interactions play a key component in delaying or preventing the onset of dementia [223, 224], while the lack thereof has often been associated with a higher risk of dementia in elderly individuals [225, 226]. The presence of social interactions presumably counteracts cognitive deterioration experienced during aging. For example, socially active older individuals were found to have a significantly lower rate of global cognitive decline compared to their infrequently active counterparts [227]. Similar results were also observed in subsequent studies, with decreased cognitive decline being associated with higher social activity [228, 229]. Furthermore, the quality of social interactions should also be taken into account. Daily social interactions, especially more pleasant social interactions, have been shown to improve cognitive function in elderly individuals [230]. A 10-year study has also demonstrated that individuals with poor quality of relationships had nearly double the risk of developing depression [231]. A further cross-sectional study in Switzerland also reported similar findings, with higher quality and frequency of social relationships being associated with lowered risk of developing clinical depression [232].

Cognitive stimulation refers to the involvement of activities generally aimed at enhancing cognitive and social capabilities. In humans, this typically involves activities that aim to stimulate thinking and memory and can come in the form of discussions, word games, puzzles, and general activities [233]. Interestingly, cognitive stimulation therapy (CST) has already been implemented as an alternative treatment method to improve the quality of life of patients with mild-moderate AD. Previous reviews have highlighted the beneficial effects of cognitive stimulation on neuropsychiatric symptoms, including depression, apathy, and anxiety [234]. Furthermore, a randomized controlled trial initiated by Carbone et al*.* also demonstrated CST as an effective strategy in combating cognitive and emotional deterioration in older patients with mild-moderate dementia [235]. In spite of this, some studies argue that despite the increase in cognitive functions, there were no observable changes in relation to mood and behavior [233, 236, 237]. Moreover, Orrell et al. also noted no significant improvements in the cognitive functions of patients with mild to moderate dementia even after 25 weeks of cognitive stimulation [238].

Implementation of EE in human models will be quite a challenging task. A procedurally uniform EE is hard to introduce for humans due to each individual’s wants and needs, and likely requires a personalized approach if this model is to succeed. Thus far, there has been only one successful implementation of EE in human test subjects. The GAIA project is a pilot study conducted in 2012 to investigate the effects of cognitive stimulation, physical activity, and socialization on patients diagnosed with AD. Specifically, the participants were involved in 1 h of mild aerobic physical exercise, 1 h of cognitive stimulation, and 30 min of social group discussion 5 days a week for 3 months. The group discovered a significant improvement in apathy, anxiety, depression, and quality of life in the active treatment group post-treatment [239]. Although promising, a larger sample size is required to draw more conclusive results. Furthermore, long-term implementation of such a program may be hard to upkeep over time due to high costs in terms of human resources and health facilities.

## Discussion and Limitations of Current Studies

The results from current studies regarding the preventive and remedial effects of EE on AD seems promising. As previously discussed, EE not only beneficially improves brain structure and function but also ameliorates behavioral deficits experienced by AD models of rodents. Albeit few, there are several studies reporting contradictory results under similar EE conditions. Presumably, these inconsistencies are not caused by the differences in EE protocols, but rather due to other experimental variables unrelated to the EE paradigm. Previously, EE has been shown to demonstrate gender-specific effects even under the same EE protocol. For instance, SD male rats exposed to 8 weeks of EE showed increased exploratory behavior towards juvenile rats, whereas no differences were found in females [240]. Furthermore, another report has noted that EE had an anxiolytic effect on male mice but had the opposite effects in female mice based on results from the elevated *T*-maze test [241]. A recent study also found EE to differentially activate neural circuits in FVB/N mice, promoting social interactions in female mice but aggressive behaviors in male mice [242]. Additionally, many studies have also noted the sex-dependent effects of EE in their respective investigations [243, 244].

The duration of EE exposure is also another factor of consideration. For instance, chronic continuous exposure to EE was found to have reduced structural changes in the hippocampus of rats compared to short and periodical exposure [245]. Furthermore, a study conducted by Nguemeni et al. noted that rats subjected to moderate exposure to EE (4 h, 8 h) had significantly increased neurogenesis compared to rats exposed to chronic EE (24 h, 48 h) [246]. Another study conducted by Singhal et al. observed that long-term EE is anxiogenic in transgenic C57BL/6 mice and affects locomotion adversely. In contrast, mice exposed to short-term EE displayed anxiolytic behaviors instead [247].

Additionally, experimenting with different models of AD may yield varying results depending on the genetic backgrounds of the model animals. Using the Tg2576 mice model as an example, no neuronal loss was observed despite having significant deposition of amyloid plaques [248]. In contrast, mice expressing multiple PS1 and APP mutations exhibit a significant neuronal loss in addition to the enhanced deposition of amyloid plaques [249]. One prominent example is when comparing the results conducted by Levi et al. and Rodriguez et al. Exposure of EE to a triple AD transgene model of mice for 6 months led to a beneficial effect on hippocampal neurogenesis [49]. In contrast, the opposite effects can be observed in transgenic apolipoprotein E (APOE) 4 mice under similar EE conditions, whereby EE not only reduced hippocampal neurogenesis but also induced cell apoptosis in APOE4 mice [250].

As discussed previously, the effects of EE are also highly age-dependent. Looking specifically at comparative studies, the effects of EE were found to be age-specific. Harburger et al. demonstrated that continuous exposure to EE for 6 weeks significantly improved spatial memory in aged males (21 months), but had no effect on young (3 months) and middle aged (15 months) C57BL/6 mice [251]. On the other hand, 3 months continuous exposure to EE was found to improve spatial memory in young Wistar male rats (21 days), but did not affect the spatial memory of their aged counterparts (7 months) [252]. In terms of AD model of rodents, early exposure to EE could function as a neuroprotective strategy against the progression of AD, while exposure of EE during mid and late-life models of AD have varying degrees of effectiveness. Presumably, this could be due to the progressive decline in neuroplasticity following aging, leading to decreased cognitive flexibility and alterations in structural plasticity [253]. Early exposure to EE plays a key part in demonstrating its effects. Deposition of Aβ starts between the age of 40–50 and precedes the onset of AD symptoms by over 20 years [254]. Presumably, the neuronal damage induced by Aβ deposition during the preclinical/early stages has yet to be significant and could be rescued by the counteracting effects of EE. On the other hand, the effectiveness of EE during the mid and late stages of AD could be diminished, due in part to the extensive neurotoxicity caused by the culmination of Aβ levels in the brain. Furthermore, exposure to EE could lead to the downstream effects of multiple molecular cascades. How EE interacts and simultaneously affects multiple downstream pathways still remains relatively unknown and could likely cause variances in results.

Although EE impacts the brain beneficially, the differences in the degree of effectiveness complicates the process of pinpointing the importance of the different EE aspects. To better understand this, it is first important to establish how different elements of EE contribute to certain effects. By far, the most unique aspect of EE is the presence of novel objects to promote cognitive stimulation. This typically comes in the form of toys, shelters, hideouts, mazes, and provision of objects of different materials, sizes, and shapes. Object rearrangement was also encouraged to further promote cognitive stimulation in rats. However, the number of objects and frequency of object rearrangement may vary according to the experimental protocol used. The effects of cognitive stimulation on the hippocampus remain relatively unclear. The establishment of EE in most studies have included both running wheels and novel objects in their experimental design. However, to fully understand the effects of cognitive stimulation through introduction of novel objects, some studies have excluded the running wheel to prevent physical enrichment induced by it. Animals exposed to novel toys have previously been shown to improve learning and memory only if the animals actively manipulated the object [255]. Similarly, animals living in an enriched with only the presence of toys had increased learning flexibility in the Morris maze test, owing to the increase in adult neurogenesis as a result of the novel environment [45]. In contrast, another study demonstrated that EE, in the absence of running wheels, failed to induce hippocampal neurogenesis and improve cognitive performance, even in the presence of a complex environment [256]. Furthermore, cognitive stimulation of adult female mice significantly affected synaptophysin levels in the neocortex and hippocampus compared to the control group [257], which could contribute to synaptogenesis in hippocampal neurons [258, 259]. However, the increase in synaptophysin did not subsequently lead to an improvement in spatial working memory based on the water radial arm maze test conducted [257]. Synaptogenesis, angiogenesis, cell proliferation and cell survival were also improved in response to being housed in a complex environment [150, 260]. Interestingly, the beneficial effects of EE also scale with the degree of enrichment, with the increased number of enrichment items corresponding to increased benefits in stereotypic behavior, behavioral measures of anxiety, growth, and stress physiology [261]. Hence, we postulate that the complexity of the cage environment and cognitive stimulation could affect memory consolidation and potentially improve synaptic plasticity. However, cognitive stimulation did not seem to contribute to hippocampal neurogenesis based on pre-existing literature.

Physical enrichment, on the other hand, has been suggested to be the most important aspect of EE based on rodent studies. The inclusion of a running wheel is often an important factor in a typically enriched cage. Furthermore, certain toys, such as ladders and stairs could also stimulate anaerobic physical activity in rodents to a certain extent. Previous studies have already discussed the neuroprotective properties of physical exercise on the hippocampus, and these include increased neurogenesis, size and volume [262], enhanced neuroplasticity [218], and promote the expression of neurotrophic factors [263, 264]. Furthermore, several studies argue that the sole presence of novel toys without the presence of running wheel is insufficient in promoting beneficial effects to the hippocampus. Mustroph et al. demonstrated that hippocampal neurogenesis were similar between male mice exposed to physical enrichment compared to mice exposed to EE. Furthermore, the group also noted that EE did not improve spatial learning based on the Morris water maze test [256]. Another study also noted the importance of physical enrichment in EE, whereby they concluded that physical exercise is the critical factor in promoting adult hippocampal neurogenesis through the release of neurotrophin factors [265]. However, it should still be noted that the presence of both physical exercise and presence of a novel environment could lead to an additive effect when promoting hippocampal neurogenesis. As reported, the group noted a 30% increase new neurons as compared to either stimulus alone [266].

The last key element being enriched in EE is the promotion of social interactions. In EE, multiple number of rodents are caged at a time, however the number of rodents per cage differs based on the protocol. The importance of the social component has led to several discussions throughout the last few decades. The presence of social interactions has previously been shown to induce hippocampal neurogenesis and improve synaptic plasticity. Lu et al. demonstrated that rats reared in group housing for 4—8 weeks had increased newborn neurons in the dentate gyrus and increased LTP in the CA1 region compared to their socially isolated counterparts [267]. Subsequently, the effects induced by social isolation could be reversed through subsequent group rearing. Similarly, the presence of social interactions was also found to improve hippocampal cell proliferation and neurogenesis, but is unable to affect learning capabilities by itself [268]. Perhaps what is considered essential in the EE paradigm is the presence of social interactions rather than the degree of social enrichment (i.e., number of animals housed per cage). For example, the effects of EE on LTP were only notable in the presence of a social environment, on the other hand, EE in socially isolated rats did not induce LTP in the hippocampus [269]. Moreover, one of the earliest studies conducted by Rosenzweig et al. suggested that social interactions alone cannot account for the cerebral effects of EE [14], with subsequent studies agreeing to this notion [191, 207, 270]. Interestingly enough, it was shown that increased group housing *(n* = 12 per cage), in the absence of physical or cognitive stimulation, could also further enhance adult hippocampal neurogenesis in adult female mice compared to the control group (*n* = 6 per cage) [271]. The presence of a social environment has been shown to improve neurogenesis and synaptic plasticity in the hippocampus. However, more studies have to be conducted to evaluate the effectiveness of social enrichment, rather than the presence of social interactions, in the EE paradigm.

Taken altogether, our understanding of current literature points to physical enrichment as the primary instigator of the effects of EE. Even so, the presence of both cognitive stimulation and social interactions are important to facilitate and to potentially further amplify these effects. However, additional studies need to be conducted to determine whether enrichment of the cognitive and social aspects of EE could improve hippocampal conditions. One potential approach is to study the effects of individual toys (ladders, stairs, plastic objects, hideouts) on brain structures which could further lead to a study investigating the combinational effects of different toys on the brain.

Despite the numerous findings of EE, comparison of effects between different studies is indeed limited. This is particularly due to the fact that the degree of enrichment for the physical, social and cognitive aspects of EE are vastly distinct between each study. For example, the amount of running wheels between certain EE protocols are different, with some studies having zero [45, 61, 272], one [46, 50, 250], or two or more running wheels [48, 270] in their study. Additionally, the amount of animals housed per group could also vary with some having up to 40 per group housing [273]. Potentially, this could create instances of overcrowding, whereby it could induce detrimental effects to the animals instead [274, 275]. Furthermore, the degree of cage of enrichment, i.e., cage size as well as the number of novel objects and the frequency of repositioning said toys also varies between studies, with the increased number of enrichment items corresponding to increased benefits [261].

Hence, in order to effectively study the effects of EE, a standardized protocol has to first be established. At this current state, it is difficult to establish a comparison of results between different EE studies due to the variance in EE protocols. The establishment of a standardized protocol not only allows the reproducibility of results to be more consistent but also allows subsequent studies to identify which variables are essential when investigating the effects of EE.

## Conclusion and Future Directions

Based on pre-existing studies, environmental enrichment presents itself as an effective neuroprotective strategy in the prevention of Alzheimer’s disease via the modulation of multiple pathways. However, how these individual pathways interact with each other during and after exposure to environmental enrichment remains relatively unknown. Future studies regarding how environmental enrichment affects multiple pathways would be essential to further understand the molecular mechanisms behind it.

Although this review has primarily focused on the effects of EE on the hippocampus. It should not be dismissed that EE also exhibits its effects via other areas of the brain. The amygdala, prefrontal cortex, and entorhinal cortex could potentially be key structures due to their susceptibility to neurodegeneration.

Implementation of EE in human models will also present itself as a rather challenging task. Although its effectiveness has been shown in animal models, the clinical application of EE in humans has yet to be widely demonstrated. Hence, it is essential to establish a better understanding of the mechanisms of environmental enrichment before it can be a viable non-pharmacological alternative for the prevention and potential treatment of AD in human patients.

## Data Availability

Not applicable.
